# Non-invasive Central and Peripheral Stimulation: New Hope for Essential Tremor?

**DOI:** 10.3389/fnins.2015.00440

**Published:** 2015-11-18

**Authors:** Moussa A. Chalah, Jean-Pascal Lefaucheur, Samar S. Ayache

**Affiliations:** ^1^EA 4391, Excitabilité Nerveuse et Thérapeutique, Université Paris-Est-CréteilCréteil, France; ^2^Service de Physiologie – Explorations Fonctionnelles, Hôpital Henri Mondor, Assistance Publique – Hôpitaux de ParisCréteil, France; ^3^Neurology Division, University Medical Center Rizk HospitalBeirut, Lebanon

**Keywords:** essential tremor, tremor, tDCS, rTMS, TBS, TENS, non-invasive brain stimulation

## Abstract

Essential tremor (ET) is among the most frequent movement disorders. It usually manifests as a postural and kinematic tremor of the arms, but may also involve the head, voice, lower limbs, and trunk. An oscillatory network has been proposed as a neural correlate of ET, and is mainly composed of the olivocerebellar system, thalamus, and motor cortex. Since pharmacological agents have limited benefits, surgical interventions like deep brain stimulation are the last-line treatment options for the most severe cases. Non-invasive brain stimulation techniques, particularly transcranial magnetic or direct current stimulation, are used to ameliorate ET. Their non-invasiveness, along with their side effects profile, makes them an appealing treatment option. In addition, peripheral stimulation has been applied in the same perspective. Hence, the aim of the present review is to shed light on the emergent use of non-invasive central and peripheral stimulation techniques in this interesting context.

## Introduction

Essential tremor (ET) is among the most frequent movement disorders in individuals above 40 years of age (Louis et al., 1995; Dogu et al., 2003). Clinically, it manifests as postural and action tremor of the arms, but may also involve the head, voice, lower limbs, and trunk (Deuschl et al., 1998; Bain et al., 2000; Elble, 2000; Raethjen and Deuschl, 2012). From an etiological perspective, it is classified as sporadic or hereditary (Kuhlenbäumer et al., 2014). Despite its high prevalence, its underlying pathophysiological mechanisms are still not well elucidated. Data from neuroimaging and neurophysiological studies have put into evidence the existence of a cerebello-thalamo-cortical (CTC) network for ET (Pinto et al., 2003; Popa et al., 2013; Hallett, 2014). The latter includes the sensorimotor cortex, olivocerebellar system, red nucleus, and thalamus (Colebatch et al., 1990; Jenkins et al., 1993; Hallett and Dubinsky, 1993; Wills et al., 1995; Bucher et al., 1997; Boecker and Brooks, 1998; Deuschl et al., 2000; Pinto et al., 2003; Raethjen et al., 2007; Quattrone et al., 2008; Shin et al., 2008; Schnitzler et al., 2009; Cerasa et al., 2010; Park et al., 2010; Bagepally et al., 2012; Paris-Robidas et al., 2012; Raethjen and Deuschl, 2012; Fang et al., 2013; Buijink et al., 2015; Choi et al., 2015; Shin et al., 2015). The presence of such a circuit was further confirmed by reports documenting ET disappearance following strokes that involved some of its components (Dupuis et al., 2010; Lim et al., 2010; Chalah et al., 2015).

Although ET is commonly thought to be a benign condition, affected patients represent a heterogeneous population (Louis, 2009) and severe cases could be very disabling (Louis, 2005). In this context, pharmacological agents have yielded modest benefits (Findley, 1987; Louis, 2000; Deuschl et al., 2011), and non-adherence to ET medications has been reported (Louis, 2015). Surgical interventions, like the deep brain stimulation, are the last-line treatment options for the most severe cases (Chopra et al., 2013). However, they have some limitations related to their cost and potential side effects (Grimaldi and Manto, 2008). Nowadays, there is a growing interest in using non-invasive central and peripheral stimulation techniques as alternatives to pharmacological and surgical interventions. Hence, in the present review, we shed light on the emergent use of these techniques in treating ET. Conversely, we excluded all data regarding invasive interventions, namely cortical, or deep brain stimulation.

## Principles of non-invasive brain stimulation techniques

In the recent years, two non-invasive brain stimulation (NIBS) techniques, i.e., repetitive transcranial magnetic stimulation (rTMS) and transcranial direct current stimulation (tDCS), gained interest for their potential implication in treating various neuropsychiatric symptoms (Kuo et al., 2014; Lefaucheur et al., 2014). These techniques are based on different principles.

To start, rTMS consists of a transcranial delivery of an electromagnetic field by a stimulation coil positioned on the patient's scalp. The induced intracortical current is strong enough to trigger action potentials according to Faraday's law of electromagnetic induction (Lefaucheur, 2012). Thus, it acts by modulating the cortical excitability in a frequency-dependent manner, as low (LF) and high (HF) stimulation frequencies (< 1 vs. > 5 Hz) have been shown to induce inhibitory and excitatory effects, respectively (Lefaucheur et al., 2014). Other than the frequency, various stimulation parameters, such as the selected cortical target, can influence the clinical effects of rTMS (Lefaucheur, 2008, 2009, 2012).

In addition to rTMS, new stimulation paradigms are being developed, of which theta burst stimulation (TBS) is the most popular (Lefaucheur, 2009). Practically, TBS consists of short bursts delivered at 5 Hz (within the theta range), each burst consisting of three magnetic pulses delivered at HF (50 Hz). TBS is either applied continuously (cTBS) for 40 s or intermittently (iTBS) during 2 s every 10 s for a total stimulation time of 200 s. Similar to rTMS, the action of TBS primarily depends on the stimulation pattern: cTBS and iTBS respectively induce long-term synaptic depression-like and potentiation-like effects, when applied over the primary motor cortex (M1) of healthy individuals (Huang et al., 2005, 2007; Teo et al., 2007; Huang, 2010; Wischnewski and Schutter, 2015).

Beside magnetic stimulation, tDCS has emerged as a promising neuromodulatory technique. It consists of delivering an electric current of low intensity (1–2 mA) over few minutes via two electrodes (anode and cathode) positioned over the scalp. By doing so, it could induce prolonged yet reversible shifts in cortical excitability and might modulate the connectivity of various neural circuits (Priori et al., 1998; Nitsche and Paulus, 2000, 2001; Priori, 2003; Nitsche et al., 2003a). The polarity of tDCS protocols determines the neurophysiological outcomes at the level of the exposed tissues: a depolarization or a hyperpolarization of the resting membrane potentials would occur following anodal or cathodal tDCS, respectively (Creutzfeldt et al., 1962; Purpura and McMurtry, 1965; Nitsche and Paulus, 2011; Paulus et al., 2013; Filmer et al., 2014). Several parameters mainly related to the electrodes properties (size, polarity, position), the used current (strength and shape), and the stimulation duration, can account for the tDCS effects (Creutzfeldt et al., 1962; Nitsche and Fregni, 2007; Nitsche et al., 2008). tDCS is safe, easily performed, well-tolerated by the patients with little or no side effects (Poreisz et al., 2007; Nitsche et al., 2008; Brunoni et al., 2012), and presents an easier and indistinguishable implementation of sham sessions compared to rTMS protocols (Gandiga et al., 2006).

## Functional underpinnings of essential tremor

Quite before their therapeutic implications, NIBS techniques have been used to explore the cortical excitability in various pathologies. For example, using transcranial electrical stimulation, one study has shown a normal central motor conduction time in four ET patients, from a series of patients with various movement disorders (Thompson et al., 1986). Using transcranial magnetic stimulation, another study has revealed normal patterns of cortical excitability in ET patients, as expressed by motor thresholds and motor evoked potentials amplitude (Romeo et al., 1998). In other works, ET patients exhibited normal patterns of intracortical inhibition (Hanajima et al., 1998; Romeo et al., 1998; Shukla et al., 2003; Chuang et al., 2014) and cerebello-thalamo-cortical inhibition (Pinto et al., 2003); the latter is a neurophysiological parameter that reflects the degree of reduction of the motor cortical output via the activation of cerebellar inhibitory projections (Pinto and Chen, 2001).

It was not until recently that cortical excitability studies have unveiled abnormal CTC functioning in patients with ET (Chuang et al., 2014; Bologna et al., 2015). Such results are of particular interest since they are in line with functional neuroimaging studies which revealed altered patterns of cortical activation and inter-regional connectivity within the CTC pathways and non-motor cortices (Wills et al., 1995; Bucher et al., 1997; Cerasa et al., 2010; Passamonti et al., 2011; Fang et al., 2013; Popa et al., 2013; Buijink et al., 2015).

In light of this evidence, the neurotransmitters imbalance has been speculated to contribute to the pathophysiology of ET. In this perspective, the glutamatergic metabolism has lately received some attention, but genetic studies have revealed controversial outcomes (Thier et al., 2012; García-Martín et al., 2013; Tan et al., 2013; Yu et al., 2013; Ross et al., 2014). The role of dopamine was also assessed in a number of studies that tried anti-psychotics (Pakkenberg and Pakkenberg, 1986; Ceravolo et al., 1999; Micheli et al., 2002; Yetimalar et al., 2003, 2005) and dopaminergic drugs (Koller, 1981; Manyam, 1984; Gironell et al., 2006) in ET management; tremor improvement was only observed in the two studies involving olanzapine (Yetimalar et al., 2003, 2005). Additionally, multidisciplinary studies are supporting the role of an aberrant GABAergic transmission in ET production (Louis, 1999; Zesiewicz et al., 2007, 2013; Boecker et al., 2010; Gironell et al., 2012; Paris-Robidas et al., 2012; Shill et al., 2012; Boecker, 2013; Helmich et al., 2013; Chuang et al., 2014; Gironell, 2014; Schneider and Deuschl, 2014). Therefore, acting on such a neurochemical imbalance might be helpful in improving ET. Interestingly, some studies have reported that NIBS after-effects take place through the modulation of the glutamatergic, GABAergic and dopaminergic transmissions (Liebetanz et al., 2002; Nitsche et al., 2003b, 2006; Stagg et al., 2009; Monte-Silva et al., 2011; Foerster et al., 2015). In addition, studies coupling NIBS techniques with functional neuroimaging have shown that rTMS, TBS, and tDCS are able to improve the functional connectivity of various cortico-subcortical networks (Bestmann et al., 2004; Grefkes et al., 2010; Eldaief et al., 2011; Keeser et al., 2011; Polanía et al., 2011, 2012a,b; Halko et al., 2014; Valchev et al., 2015).

Taken together, these data suggest that NIBS techniques would ameliorate ET by (i) acting on the neurochemical imbalance at the site of stimulation, (ii) subsequently modulating the local cortical excitability and by doing so, (iii) restoring the functional integrity of the CTC network of ET.

## NIBS studies and essential tremor

Early neurophysiological studies have assessed the role of NIBS techniques in modulating the physiological parameters in ET. Single-pulse TMS over M1 was successively able to reset ongoing tremor activity (Britton et al., 1993a; Pascual-Leone et al., 1994; Yu et al., 2001). In a recent study, ET resetting resulted from applying single-pulse or paired-pulse TMS over M1 or the supplementary motor area (SMA), but not over the cerebellum (Lu et al., 2015).

Electrical stimulation was also used in the same setting. Although early reports have documented a failure of transcranial motor electrical stimulation in resetting ET (Pascual-Leone et al., 1994), a new study have provided evidence regarding the ability of transcranial alternating current stimulation to induce tremor entrainment when applied over the cerebellum of ET patients (Brittain et al., 2015).

These data altogether have pushed the research toward studying the possible therapeutic implementations of NIBS in terms of ET. A PubMed search using the keywords rTMS/TBS/tDCS and essential tremor has identified seven English papers. Their designs and outcomes are reported in Table 1.

**Table 1:** **Non-invasive brain stimulation studies in essential tremor**.

**References**	**Population**	**Intervention**	**Measured outcome**	**Results**
Gironell et al., 2002	10 ET patients	Single session of active or sham 1 Hz rTMS over the cerebellum (on the midline, 2 cm below inion) separated by 1 week interval	Clinical (TCRS) and accelerometric evaluation before (−5 min), immediately after (+5 min), and 1 h after (+60 min) each session	Short-term clinical and accelerometric improvement, disappearing within 5 min after the end of the active sessio
		Each session: 20 min, 300 pulses, 100% of the maximum output intensity		
Avanzino et al., 2009	15 ET patients vs. 11 HCs	Comparing the motor behavior of both groups during repetitive finger tapping movements of the right hand by the means of a sensor-engineered glove	Timing properties and motor behavior	Longer TD, lower ITI, and increased ITICV in ET patients compared to HCs
	11 ET	One session of active or sham^†^ 1 Hz rTMS over the right ipsilateral cerebellum (3 cm lateral and 1 cm beneath the inion) separated by at least 2 week interval		Transient reduction of TD values and normalization of ITI and ITICV values
		Each session: 10 min, 600 pulses, 90% of RMT		No effects of rTMS on the frequency and intensity of tremor
Popa et al., 2013	11 ET patients vs. 11 HCs	5 consecutive daily active sessions of neuronavigated bilateral 1 Hz rTMS over the posterior cerebellar cortex (targeting lobule VIII of each cerebellar hemisphere)	Rs-FC of the CTC network and DBN (as control) before and after rTMS (day 1 and 5)	Improvement in rs-FC within CTC network, but not within DBN
		Each session: 15 min, 900 pulses, 90% of the RMT	Clinical (FTM) and neurophysiological (electromyographic and accelerometric) tremor assessment at baseline, day 5, day 12, and day 29 after the last session	Long-term effects lasting for 3 weeks after the last session, consisting in clinical scores improvement and a reduction in tremor amplitude (but not frequency)
Hellriegel et al., 2012	10 ET patients vs. 10 HCs	2 cTBS sessions: one real (80% of AMT), one control (30 % of AMT) over the left hand motor area separated by at least 1 week interval	Corticospinal excitability parameter	Reduction of corticospinal excitability in the stimulated M1 following real cTBS in HCs, but not in ET patients
		Each session: Two 20-s trains with inter-train interval of 1 min, bursts being repeated every 200 ms	Clinical (FTM) and quantitative (accelerometric) rating of tremor before and at 10, 25, and 40 min after cTBS	Reduction in tremor amplitude, but not frequency following real cTBS, lasting for at least 45 min
				No significant clinical reduction of ET after real cTBS
Chuang et al., 2014	13 ET patients vs. 18 HCs	3 cTBS sessions: 2 real (80% of AMT) over the left M1 or PM and 1 sham^£^ (60% of AMT, flipped coil) over M1) separated by at least 1 week	Excitability parameters (SICI, CSP, ICF)	Reduced cTBS suppressive effect on motor cortical excitability in ET patients compared with HCs
		Each session: One 40 s-train, bursts being repeated every 200 ms	Accelerometric tremor recording before and 22–25 min after cTBS	Reduction in tremor amplitude, but not frequency following motor, premotor, but not sham session
Bologna et al., 2015	16 ET patients vs. 11 HCs	2 cTBS sessions: one real (80% of AMT) over the right cerebellar hemisphere (3 cm laterally to and 1 cm below the inion) and one sham over the neck muscles separated by at least 1 week interval	Excitability parameters (input/output curve)	Reduced cTBS suppressive effect on motor cortical excitability in ET patients compared with HCs
		Each session: One 40 s-train, bursts being repeated every 200 ms	Assessment of tremor and reaching movements at baseline, and at 5 and 45 min after cTBS	No significant change in tremor severity and reaching movements after any session
Gironell et al., 2014	10 ET patients	10 consecutive daily sessions of either active or sham-cathodal cerebellar tDCS separated by a 3-month wash-out period. (two cathodal electrodes placed symmetrically over both cerebellar hemispheres, 3 cm lateral to the inion; and two anodal electrodes positioned over Fp1 and Fp2 EEG leads position)	Clinical (TCRS) and accelerometric tremor evaluation before (at day 1), during (10 min after onset) and 60 min after the first session	No significant acute or long-lasting tDCS effects in any outcome measure
			Clinical (TCRS) and accelerometric tremor assessment; and disability scale evaluation before the first session, and at day 10 and 40 after the last session	
		Each session: 20 min; 2 mA		

*AMT, active motor threshold; CSP, cortical silent period; cTBS, continuous theta burst stimulation; CTC, cerebello-thalamo-cortical; DBN, default brain network; ET, essential tremor; FTM, Fahn Tolosa Marin tremor rating scale; HCs, healthy controls; ICF, intracortical facilitation; ITI, inter tapping interval; ITICV, coefficient of variation of the inter tapping interval; MEPs, Motor evoked potentials; Min, minutes; M1, primary motor cortex; PM, premotor cortex; RMT, resting motor threshold; rs-FC, resting state functional connectivity; rTMS, Repetitive transcranial magnetic stimulation; SICI, short interval intracortical inhibition; TCRS, Tremor clinical rating scale; TD, touch duration; tDCS, Transcranial direct current stimulation*.

† *Sham design was undescribed and performed in seven patients only*.

£ *Sham session was performed in 10 patients only*.

### rTMS and essential tremor

The first published study involved 10 patients with ET of the upper limbs, in a double-blind, crossover, and sham-controlled design (Gironell et al., 2002). The patients received two sessions of either active or sham 1 Hz rTMS over the cerebellum separated by 1-week free interval. Compared to sham, significant short-term effects were observed following real rTMS session, as expressed by the improvement of the tremor clinical rating scale and tremor frequency on accelerometric studies. However, such an improvement did not last more than 5 min following the session.

In a second study, 11 ET patients underwent a single session of 1 Hz rTMS over the cerebellum to evaluate the potential modulation of motor behavior during repetitive finger tapping movements of the right hand using a sensor-engineered glove (Avanzino et al., 2009). Seven patients also received sham session at least 2 weeks apart from the real one. Compared to healthy controls, ET patients presented lower inter-tapping interval (ITI), increased coefficient of variation of ITI (ITICV), and longer touch duration (TD). The latter represents the time when the thumb and another finger are in contact, before their separation which results in generation of the rhythmic movement. It is probably the sum of the time required for the thumb to get an adequate perception of another touched finger (sensory time), and the time needed to plan for the next movement (preparatory time), and by doing so, to maintain the rhythmic tapping (Georgiou et al., 1995). The cerebellum participates in the timing of movement and sensation (Rao et al., 2001), and increased ITICV was previously reported in the context of ET (Farkas et al., 2006). In the absence of sensory deficits in ET patients (Nahab et al., 2007), the abnormal TD values hint toward pathological phenomena at the level of sensorimotor integration, where the sensory information is used for the initiation of motor planning (Avanzino et al., 2009). In this study, rTMS reduced the TD values and normalized the ITI/ITICV values in ET patients in a transient manner. However, in contrast to the first study by Gironell et al. (2002), 1 Hz rTMS was unable to modify the frequency or the intensity of ET, which might be explained in part by the lower stimulation intensity adopted in this study.

A third study included eleven ET patients and eleven healthy controls (Popa et al., 2013). Here, the resting-state functional connectivity (rs-FC) of the CTC circuits and default brain network (DBN) was assessed before and after the application of five consecutive daily sessions of 1 Hz rTMS over the posterior cerebellar cortex. Stimulation was performed using a neuronavigation system to target the lobule VIII of both cerebellar hemispheres. Tremor was assessed using clinical scales and accelerometric recordings. A significant improvement was observed on clinical scales, and was associated with a reduction in tremor amplitude, but not frequency. This improvement persisted for 3 weeks after the last rTMS session and was associated with a near-normal restoration of the connectivity within the CTC network, but not within the DBN. These findings could reflect pronounced neuroplasticity effects that might have resulted from the repetition of the stimulation sessions. In addition, unlike the two previous studies, this one adopted a neuronavigation-guided paradigm, which might have an important role in optimizing rTMS protocols (Lefaucheur, 2010).

### TBS and essential tremor

Two studies have applied cTBS over the motor and premotor cortices which are the key elements for movement preparation, selection, and execution. The first one assessed the effects of cTBS on tremor and cortical excitability in 10 patients with ET and 10 healthy controls (Hellriegel et al., 2012). Each participant randomly received two sessions of real or control cTBS over the left hand motor area separated by at least 1 week. A subclinical reduction in tremor amplitude, but not frequency, was observed following real cTBS session and lasted for at least 45 min. Hereby, the absence of significant clinical improvement could be justified by the logarithmic relationship between accelerometric and clinical tremor assessment (Elble et al., 2006). In line with the first study, a second cTBS study has found an exclusive reduction in tremor amplitude following cTBS delivered to the motor and premotor cortices in 13 patients with ET (Chuang et al., 2014).

Interestingly, in both studies, motor cortical, or corticospinal excitability was assessed using different variables (Table 1); and it was shown that the suppressive cTBS effects on cortical excitability was either reduced or absent in ET patients compared to healthy controls. This suggests that the observed improvement in tremor amplitude appears to be independent of the modulation of the corticospinal motor output. Such observation is in accordance with recent evidence hinting toward the occurrence of cTBS-induced behavioral or rs-FC changes, unrelated to those of cortical excitability (Silvanto et al., 2007; Gentner et al., 2008; Nettekoven et al., 2014).

The third cTBS study was a randomized, sham-controlled, double-blind study that assessed the effects of right cerebellar cTBS in ET patients and healthy controls (Bologna et al., 2015). The authors did not find any effect of cTBS on clinical or kinematic measures of tremor. However, as in the two previously published trials, the suppressive effects of cTBS on cortical excitability were lost in ET patients compared to their healthy counterparts.

### tDCS and essential tremor

Similar to rTMS, the ability of tDCS to modulate the cerebellar excitability has been previously reported (Galea et al., 2009). Gironell and colleagues have studied the effects of cathodal cerebellar tDCS in ten patients with ET (Gironell et al., 2014). Each patient randomly received two blocks, each consisting of 10 consecutive sessions of either active or sham bilateral cerebellar tDCS separated by at least 3 months of washout interval. Clinical and accelerometric studies did not reveal any short-term or long-term benefits following the real tDCS sessions. However, this study suffers from some limitations related to the small sample size and the high intra-subject variability of accelerometric measures.

### Peripheral stimulation and essential tremor

Besides trying to act at the level of the central oscillators, an alternative would be to focus on the ET substrates in charge of transmitting and displaying the symptom, namely the peripheral nerves and muscles. The efficacy of symptomatic interventions was tested in tremulous patients regardless of the tremor origin.

### Transcutaneous electrical nerve stimulation

Transcutaneous electrical nerve stimulation (TENS) is a non-invasive, cheap, and safe technique that consists of delivering an electrical current at various frequencies, intensities and pulse duration on a limited skin surface (Sluka and Walsh, 2003; Lim et al., 2010). TENS can modulate motor cortex excitability by acting on the sensory afferent input and the sensorimotor integration at the cortical level (Tinazzi et al., 2005a). In clinical practice, TENS is mainly applied to treat pain syndromes of various origins. In the field of movement disorders, TENS was also found to have some efficacy in dystonic tremor (Bending and Cleeves, 1990), writer's cramp dystonia (Tinazzi et al., 2005b, 2006), and psychogenic movement disorders (Ferrara et al., 2011).

As for ET, the first study to assess the effects of peripheral nerve stimulation was reported by Britton et al. (1993b). Here, the application of supramaximal median nerve shocks at the elbow (0.5 ms square-wave electrical pulse applied as five stimuli, randomly delivered at 5–8 s of interval; with sufficient intensity able to produce maximal EMG responses at the flexor carpi radialis) was able to cause acute inhibition, then synchronization of the EMG activity in 10 patients with ET, nine patients with Parkinson's disease (PD) tremor and nine healthy controls mimicking wrist tremor.

In another study, Munhoz and colleagues assessed TENS effects in five patients with ET and two patients suffering from tremor attributed to peripheral neuropathies (Munhoz et al., 2003). For this purpose, the cathode was placed over the brachial plexus with the reference electrode over the C7 spinous process. A 15-min stimulation was performed, using different settings (frequencies: 5, 10, 50, and 100 Hz; one side vs. the other side vs. both sides simultaneously). No significant improvement was observed at any endpoint (accelerometric variables, tremor rating, and self-reported impression scales) (Munhoz et al., 2003).

### Functional electrical stimulation

Another alternative to alleviate tremor would be through performing muscular contraction either voluntarily (Dietz et al., 1974; Héroux et al., 2010), or through neurostimulation using the so-called “closed-loop functional electrical stimulation” (FES) (Elek and Prochazka, 1989; Javidan et al., 1990, 1992; Prochazka et al., 1992; Gillard et al., 1999). The earliest study was performed by Javidan and colleagues and involved three patients with ET, four patients with PD and six patients with multiple sclerosis (MS) suffering from cerebellar tremor (Javidan et al., 1992). The authors documented attenuation in tremor amplitude by 73% in ET, 62% in PD, and 38% in MS. Interestingly, a minor shift in tremor frequency was observed in MS group, without any changes in ET or PD patients.

In an attempt to counteract tremor, this method consists of monitoring joint displacement using a miniature displacement transducer. The next step is to use the signals acquired from the joint angle excursions to perform an out-of-phase stimulation, in order to activate the antagonist muscle during involuntary activation of the agonist one. Counteracting tremor is possible via a feedback filter with bandpass characteristics designed to selectively attenuate tremor (2–5 Hz) while barely affecting the slow voluntary movements. Unfortunately, such a technique has some limitations. For instance, the FES ability to act on a given antagonistic pair restricts its role in patients with ET where the symptom is often multidirectional and involves multiple joints. In addition, there is still uncertainty regarding the optimal way of electrodes positioning aiming to stimulate specific muscle groups. Moreover, despite the positive outcome of the preliminary study by Javidan et al. (1992), there is an intra-individual variation in tremor amplitude and frequency, which might limit the efficacy of FES to a specific frequency range and hence requires repeated calibrations of the feedback filter (Javidan et al., 1992). Furthermore, a certain degree of discomfort and fatigue might result from applying the phasic electrical stimulation, which makes the technique less appropriate for daily life usage. In this view, many FES studies have suggested some solutions to circumvent the faced difficulties.

One of the studies tried to explore if the type of feedback filter might affect the clinical outcome (Gillard et al., 1999). For example, a digital filter was found to be superior to its analog counterpart in terms of tremor attenuation in PD patients (84 vs. 65%, respectively). Other studies proposed that a way to improve the FES system would be by implementing a control algorithm that chiefly relies on feedback from inertial sensors and EMG (Zhang and Ang, 2007; Bó et al., 2008; Widjaja et al., 2009; Rocon et al., 2010). This issue was further addressed by a group of authors who applied a new FES system consisting of hardware and software, in three ET patients, four PD patients and five healthy controls (Popović Maneski et al., 2011). In this system, two gyroscopes served the purpose of inertial sensors that provided real-time estimation of tremor, the data of which being digitized and delivered to a computer system that implements an algorithm mainly relying on a Butterworth second-order adaptive bandpass filter (Popović et al., 2010). Via a high-speed USB, the computer controls a battery-driven programmable multichannel stimulator that supports asynchronous activation of several electrodes. The latter are located over the dorsal and volar sides of the forearm, and perform a specific out-of-phase stimulation. The experimental protocol on healthy controls has proven its efficiency in activating the antagonistic muscles in a strong and asynchronous manner, which could not be voluntarily suppressed by the individuals. The intervention was beneficial in only two of the three ET patients. The current design aimed to control several upper extremity joints (fingers, wrist, elbow, and shoulder) and thus was able to overcome the limitations of the mono-joint design discussed in the first work (Javidan et al., 1992). Furthermore, it permitted the stimulation of one muscle group using multi-pad electrodes rather than the previously used single cathode, which could ensure the selectivity (Popović-Bijelić et al., 2005; Popović and Popović, 2009; Popovic et al., 2009; Malešević et al., 2010b) and decrease the stimulation-induced fatigue (Popovic and Malesevic, 2009; Malešević et al., 2010a,b). Following the same principles, other studies combined FES with a brain-computer interface (Grimaldi and Manto, 2010; Rocon et al., 2010). This allows for a multimodal detection of the movement intentionality by fusing signals from EEG, EMG and kinematic sensors (in particular gyroscopes and accelerometers). Another group of authors has relied on EMG detection combined with an iterative Hilbert transform to apply FES in six patients with ET or PD (Dosen et al., 2015). In this study, the tremor was reduced by 46–81 and 35–48% when using the motor and sensory stimulation, respectively, in five of the six studied patients. Thus, using electrical stimulation below motor threshold seems to be more effective than the sensory one, and prevents muscle fatigue and discomfort.

Finally, fixed-intensity FES was suggested as an alternative to the classical closed-loop FES (Bó et al., 2014). The rationale was that fixing the intensity might make the intervention more comfortable and accepted by patients. Keeping in mind that in ET, the tremor propagates from proximal to peripheral joints, this technique is intrinsically stabilizing compared to the antiphase FES stimulation, where an unstable proximal performance might increase the distal tremor. A single session of fixed-intensity FES was applied to the wrist or fingers of 10 ET patients (pulse width: 150 μs; frequency: 40 Hz; with manual regulation of stimulation intensity respecting patient's feeling of discomfort). The system was similar to the previous ones in a way that it relies on inertial sensors (gyroscopes and accelerometer) and high-pass filter. Tremor was suppressed in eight patients, did not significantly change in one patient, and was exacerbated in one of them.

## Conclusion

Although only few data are available, some of the preliminary results would pave the way for future studies on a larger scale.

Concerning NIBS, the discrepancy encountered in the results could arise from many factors. On the one hand, all of these studies had assessed the effects of different NIBS techniques in small samples (ranging from 10 to 16 ET patients), and adopted different number of sessions (ranging from 1 to 10 consecutive daily sessions). On the other hand, the fact that ET patients represent a heterogeneous population with regard to the functional brain topography, tremor site, and severity, age of onset, disease duration, pharmacological interventions at the time of protocol, and the patients' sensibility to these treatments, can partly explain the subsequent variation in response to the performed NIBS interventions. In fact, the variation in pre-interventional brain connectivity or genetic polymorphisms in neurotrophic factors can influence NIBS effects (Antal et al., 2010; Cárdenas-Morales et al., 2014; Nettekoven et al., 2014).

Therefore, improving the outcome of NIBS techniques in ET patients can be obtained by acting on different parameters, such as rTMS frequency, TBS pattern, or tDCS polarity. Particularly, increasing the duration or the number of stimulation sessions might enhance the therapeutic effect to a meaningful clinical level, based on the likely dose-dependent effects of these interventions (Nettekoven et al., 2014). Moreover, considering the different functional topography seen in ET patients, a smart attempt to optimize NIBS protocols would be by individualizing them. This could be achieved by performing a baseline functional neuroimaging and neurophysiological interventions in each patient. This approach would improve the definition of the optimal NIBS targets for image-guided procedures. Furthermore, future studies should not be limited to targeting M1 or the cerebellum, but rather should assess the potential value of other targets in terms of motor or cognitive improvement.

Besides acting on the disturbed central networks, peripheral stimulation constitutes a symptomatic approach that proved some benefits in ET. FES can significantly improve tremor of various etiologies, but its use is limited by its practical and esthetic profile. Finally, concerning TENS techniques, only preliminary data are available and further studies are required before drawing any conclusion.

### Conflict of interest statement

The authors declare that the research was conducted in the absence of any commercial or financial relationships that could be construed as a potential conflict of interest.
